# Case Report: A rare cause of pancreatitis - fish bone

**DOI:** 10.3389/fmed.2025.1645292

**Published:** 2025-11-14

**Authors:** Yifeng Wei, Ying Qian, Weimei He

**Affiliations:** 1 Huzhou Central Hospital, Fifth School of Clinical Medicine of Zhejiang Chinese Medical University, Huzhou, China; 2 Huzhou Central Hospital, Affiliated Central Hospital of Huzhou University, Huzhou, China; 3 Community Health Service Center of Dipu, Anji, Huzhou, China

**Keywords:** fish bone, pancreatitis, foreign body, perforation, endoscopic removal

## Abstract

There is a wide variety of causes of pancreatitis, such as cholelithiasis and ethanol, of which only a small percentage are induced by foreign bodies, while cases of pancreatitis due to accidental ingestion of fish bone are seldom reported. Most accidentally ingested fish bones are eliminated through the digestive tract. It is very rare for them to penetrate the gastrointestinal tract and enter the pancreas, causing related complications. Here, we present a case of pancreatitis secondary to accidental ingestion of a fish bone. The case was initially poorly managed with medical treatment and further imaging suggestive of perforation and infection, which was successfully removed after exploratory laparotomy and intraoperative gastroscopy. A history of accidental fish bone ingestion is important and that contrast-enhanced computed tomography is indispensable for an early diagnosis. Endoscopic removal has the advantages of being less invasive, less painful, and easier to perform and is therefore preferred in most patients in recent years.

## 1 Introduction

Acute pancreatitis (AP), affecting approximately 34 per 100,000 individuals annually, is predominantly caused by gallstones, alcohol, or hyperlipidemia (1, 2). Foreign body-induced AP, however, accounts for <1% of cases, with fish bone penetration into the pancreas being exceptionally rare (3). Accidental ingestion of fish bones is common in life, most of which are passed from the gastrointestinal tract on their own, while a small number of them will penetrate the intestinal wall and enter neighboring organs, causing inflammation or abscess formation (4). Most ingested fish bones pass uneventfully through the gastrointestinal tract; perforation occurs in <1%, and pancreatic involvement is scarcely documented (5, 6). Here, we present a case of fish bone-induced pancreatitis in a patient with Billroth II anatomy, emphasizing diagnostic pitfalls and management strategies.

## 2 Case presentation

**Figure 1:**
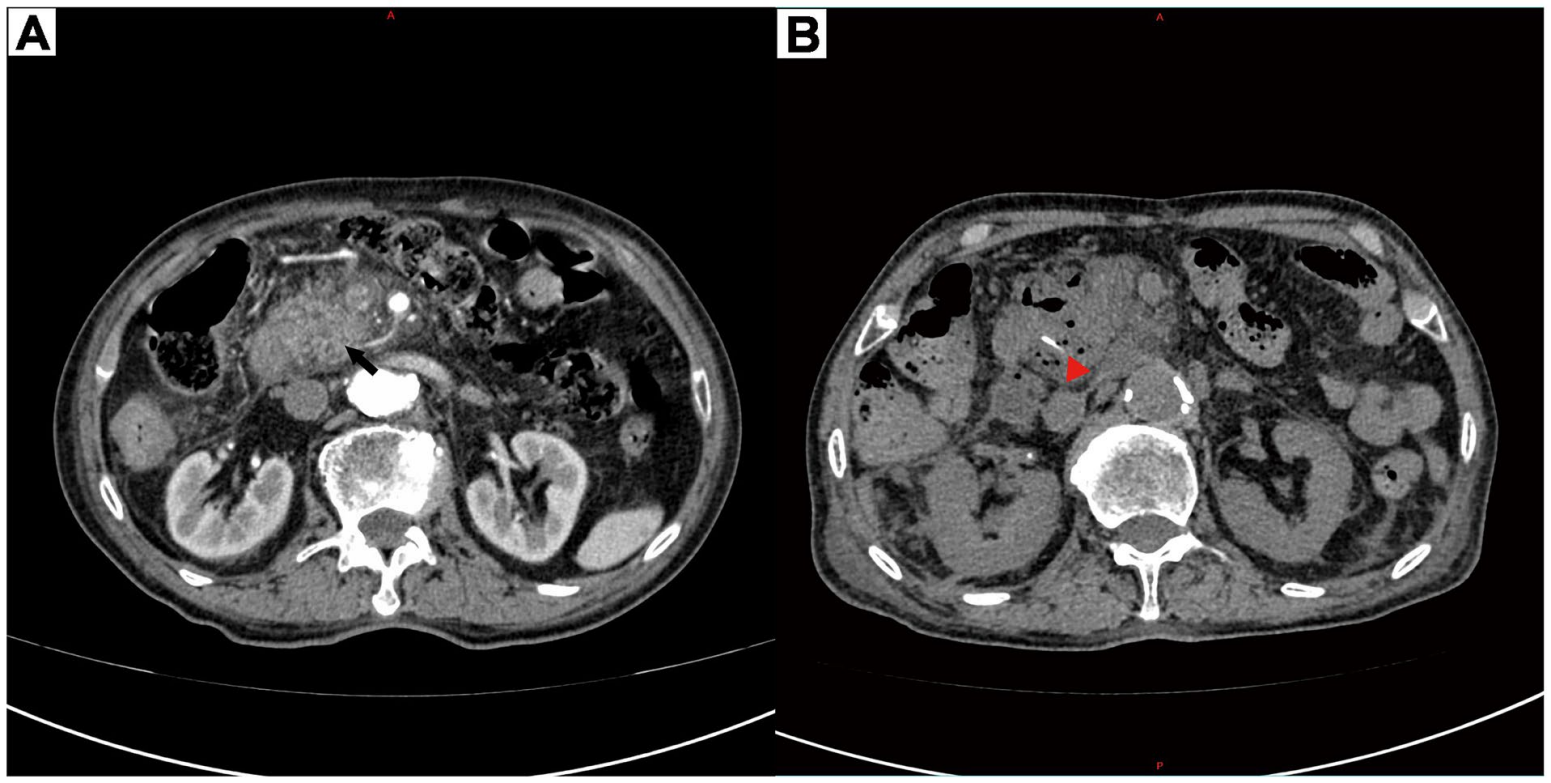
**(A)** Contrast-enhanced computed tomography showed fullness of the head of the pancreas, peripheral oozing, and blurring of the surrounding fat space (black arrow). **(B)** Contrast-enhanced computed tomography showed a localized hyperdense shadow (red triangle) in the head-duodenal region of the pancreas with a long diameter of approximately 21 mm and a fixed morphology.

An 85-year-old man presented with 3 days of severe epigastric pain, nausea, and fever (38.3 °C). The abdominal pain was of a severe degree, not self-relieving. The patient has hypertension and Meniere’s disease, as well as a history of cerebral infarction. He denied alcohol consumption or gallstone history but had undergone a Billroth II gastrojejunostomy for a gastric ulcer more than 40 years ago. Physical examination revealed fever up to 38.3 °C, with pressure and rebound tenderness on palpation of the upper abdomen. Laboratory findings revealed elevated leukocytosis (13.8 × 10^9^/L) and CRP (119.4 mg/L), but normal serum amylase (58 U/L) (Table 1). Abdominal contrast-enhanced computed tomography (CECT) demonstrated pancreatic head edema and peripancreatic exudation (Figure 1A). No biliary obstruction was observed. Consequently, the diagnosis of acute pancreatitis was made. After admission to the hospital, the patient was fasted, given gastrointestinal decompression, acid suppression with proton pump inhibitors, inhibition of pancreatic secretion with growth inhibitors, anti-infection with antibiotics, and started on intravenous fluids.

**Table 1:** Blood test results on admission.

Test	Result	Reference range
White blood cell count, 10^9^/L	13.8	3.5–9.5
Neutrophil, 10^9^/L	12.6	1.8–6.3
Hemoglobin, g/L	104.0	130.0–175.0
Platelet count, 10^9^/L	173.0	125.0–350.0
High-sensitivity C-reactive protein, mg/L	119.4	< 10.0
Amylase, U/L	58.0	20.0–125.0
Procalcitonin, ng/mL	0.46	< 0.065

**Figure 2:**
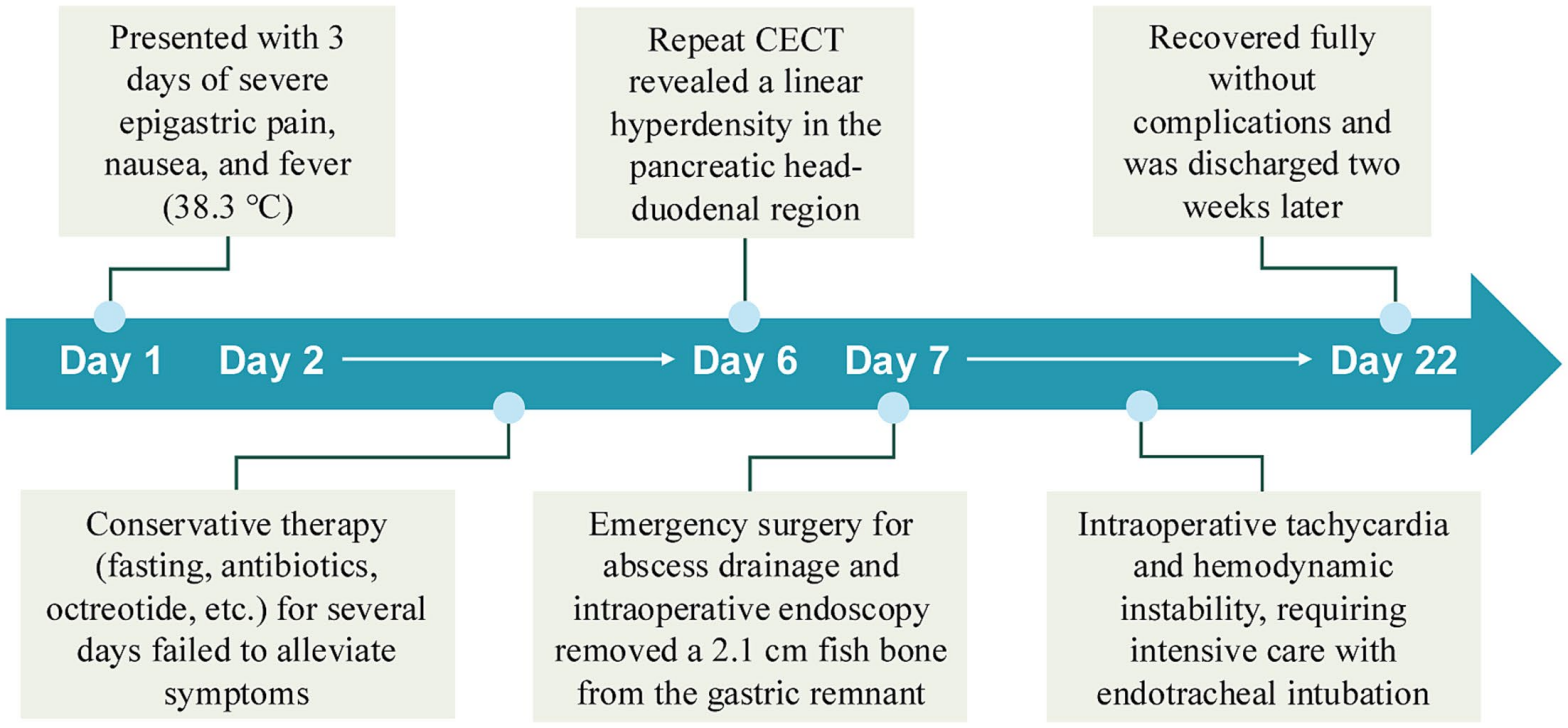
Timeline of clinical presentation and management.

However, after several days of treatment, initial conservative therapy (fasting, antibiotics, octreotide) failed to alleviate the symptoms. Although the abdominal pain persisted, the blood and urine amylase levels were still not high. Repeat CECT revealed a linear hyperdensity shadow in the pancreatic head-duodenal region (Figure 1B). The patient then underwent an emergency laparotomy and was classified as ASA IIIE according to the American Society of Anesthesiologists (ASA) Physical Status Classification System. Emergency surgery revealed infection around the pancreatic head and duodenum. Despite abscess drainage, no foreign body was initially detected. Intraoperative endoscopy was subsequently performed, and a 2.1 cm long fish bone was identified in the gastric pouch, which was successfully removed with foreign body forceps. The patient experienced intraoperative tachycardia and hemodynamic instability, requiring postoperative intensive care with endotracheal intubation for further life support. Two weeks later, with stable vital signs and no surgical complications, he made a full recovery and was successfully discharged from the hospital. Figure 2 shows the timeline of clinical presentation and treatment during hospitalization.

## 3 Discussion

Acute pancreatitis is a common clinical disorder of the digestive system, usually caused by abnormal activation of pancreatic enzymes and self-digestion of pancreatic tissue. It has a wide range of causes. Globally, the most common causes in patients with acute pancreatitis are cholelithiasis, alcohol and hyperlipidemia (2). An uncommon cause of pancreatitis is foreign body-induced pancreatitis.

The diagnosis of acute pancreatitis needs to be established on two of the following three items: characteristic abdominal pain, elevated blood amylase or lipase, and typical imaging changes. If a patient has a presentation of abdominal pain suggestive of acute pancreatitis, but serum amylase and/or lipase activity is less than three times the upper limit of normal, imaging studies are required to confirm the diagnosis (7, 8). Severe pancreatic necrosis or microcirculatory impairment may limit enzyme production and release, explaining the discordance between clinical and biochemical findings (9, 10). The delay in patient visits also connected with the decline in amylase levels to some extent (serum amylase levels often rise within hours of the onset of symptoms and revert to normal within three to five days) (11, 12). Diagnostic imaging is crucial in individuals with little or no enzyme elevation (13). CECT is the most effective imaging technique to assess the severity of AP, distinguish other serious abdominal conditions, and find complications. In addition, CECT can determine the presence of pancreatic necrosis and provide clues to the etiology of AP, which is an important clinical guideline for subsequent treatment (7).

Foreign body-induced AP is clinically uncommon. Fish bones are one of the most common foreign bodies causing perforation, mostly seen in cultures where unfilleted fish is a culinary delicacy (14). For instance, people in some Asian countries tend to prefer freshwater fish with numerous small bones. However, cases of fish bones penetrating the gastrointestinal tract and causing pancreatitis are very rare, with only less than 20 cases reported to date (4). A systematic analysis of 16 cases (Table 2) revealed: median age 65 years (range: 32–85), 56% male; 100% cases relied on CT, while only 19% exhibited elevated amylase; The prognosis for those who underwent surgery in time was mostly favorable; Endoscopic/laparoscopic approaches increased post-2018, reflecting a shift toward minimally invasive techniques.

**Table 2:** Reported cases of pancreatic injury induced by accidental ingestion of fish bone.

Author	Patient details	Chief complaint	Pancreatic enzyme levels	Diagnosis	Fish bone size (cm)	Site of perforation	Treatment
Goh et al. (20)	60 Y/O, F	Epigastric discomfort for 2 weeks	N/A	CT	3.0	Stomach	Laparotomy
Goh et al. (21)	32 Y/O, M	Fever with chills and rigors for 5 days	−	CT	3.0	Duodenum	Laparotomy
Wang et al. (22)	68 Y/O, M	Dull pain over the epigastric area for 4 weeks	−	CT	2.3	Stomach	Laparotomy
Chiu et al. (3)	66 Y/O, F	Fever and chills, epigastric pain, and body weight loss of about 3 kilograms for 2 weeks	+	CT	N/A	Stomach	Laparotomy
Yasuda et al. (23)	73 Y/O, F	Persistent upper abdominal dull pain for 3 days	−	CT	4.0	Duodenum	Laparotomy
Symeonidis et al. (24)	57 Y/O, F	Midepigastric pain, nausea, and vomiting for 1 day	+	Contrast-enhanced CT	N/A	Duodenum	Laparotomy
Huang et al. (6)	53 Y/O, F	Intermittent, dull epigastric pain for 3 months, worsening abdominal pain and fever for 2 days	N/A	CT	3.2	Stomach	Laparotomy
Wang et al. (25)	65 Y/O, M	Recurrent upper abdominal pain and gradual weight loss for 2 months	−	CT	3.0	Stomach	Laparotomy
Mima et al. (26)	80 Y/O, M	Epigastric pain for 1 day	−	CT	2.5	Stomach	Laparoscopic surgery
Xie et al. (27)	32 Y/O, M	Abdominal pain over 5 days	−	Endoscopic ultrasonography and CT	3.5	Stomach	Laparoscopic surgery
Attila et al. (28)	76 Y/O, M	Epigastric pain for 2 days	N/A	CT	3.5	Duodenum	Endoscopic removal
Mulita et al. (29)	59 Y/O, F	Fever and epigastric pain for 2 days	−	CT	3.0	Stomach	Laparoscopic surgery
Wang et al. (30)	67 Y/O, M	Abdominal pain over 3 months	−	Bone condition CT	3.2	Stomach	Laparoscopic surgery
Sharma et al. (14)	64 Y/O, M	Epigastric pain for two days	+	CT	3.3	Stomach	Endoscopic removal
Wu et al. (4)	49 Y/O, F	Worsening dull epigastric pain for over 2 months, general soreness, and fever for 2 days	−	Contrast-enhanced CT	4	Stomach	Laparoscopic surgery
Our case	85 Y/O, M	Epigastric pain for 3 days	−	Contrast-enhanced CT	2.1	Stomach	Endoscopic removal

M, male; F, female; +, outside normal limits; −, within normal limits.

In our case, altered anatomy (Billroth II anatomy) may predispose to fish bone retention and migration. Post-surgical adhesions likely facilitated transmural penetration into the pancreas. In addition, fish bone migration as a physical trauma leads to parenchymal cell damage in the pancreas, where intracellular contents, including damage-associated molecular patterns, are released into the extracellular area and trigger a local inflammatory cascade (e.g., TLR4/NF-κB activation) by binding to Toll-like receptors and other pattern recognition receptors, further leading to pancreatic parenchymal injury and abscess formation (15–17). Moreover, depending on the trajectory of the foreign body, the fish bone can be located in different organs, including the small intestine, retroperitoneum, and liver. For instance, a case of an ingested fish bone at the caudate lobe has also been described (18).

Medical treatment of acute pancreatitis includes fluid resuscitation, reduction of pancreatic fluid secretion, and control of inflammation. If conservative treatment is not effective, the cause of the disease should be found and removed as early as possible by endoscopy or surgery. Once a foreign body is diagnosed, such as a fish bone, the choice of laparotomy or laparoscopic surgery or endoscopic treatment depends on the length, location, and complications of the fish bone. Endoscopic removal has the advantages of being less invasive, less painful, and easier to perform and is therefore preferred in most patients. A small percentage of patients may develop complications such as abdominal abscesses and require surgery. Laparoscopy also offers a magnified view and better visualization of the lesser sac, which could lead to less invasive approaches, especially for high-risk patients such as an 85-year-old patient. Probably, this should be the first approach, but the patient had previous upper gastrointestinal surgery, and potentially there were adhesions in the area. If there is evidence that part of the hyperdense foreign body is in the lumen of the stomach, then obviously an endoscopic removal should be the optimal approach. In certain circumstances, effective drainage and infection control can also facilitate patient recovery when a fish bone is not easily accessible, as it may become encapsulated by human tissue and remain dormant. Katsuhiko Horii et al. documented a case of a hepatic abscess resulting from fish bone penetration. Percutaneous abscess drainage was performed under ultrasonographic guidance. Following drainage, the patient became afebrile, and imaging findings demonstrated that the abscess cavity had decreased in size (19). It is worth noting that if minimally invasive drainage proves insufficient, surgery serves as the definitive treatment.

## 4 Conclusion

When we see a patient with normal amylase AP, we should consider not only whether it is severe pancreatitis, but also whether it is caused by alcoholic, hypertriglyceridemic or a foreign body, such as the fish bone mentioned in this case. A history of accidental ingestion of fish bone is important, and CECT is indispensable for early diagnosis. Endoscopic removal, when feasible, offers a safer alternative to laparotomy.

## Data Availability

The original contributions presented in the study are included in the article/supplementary material, further inquiries can be directed to the corresponding author.
